# Reversible Binding of Nitric Oxide in a Cu(II)-Containing Microporous Metal-Organic Framework

**DOI:** 10.3390/molecules30143007

**Published:** 2025-07-17

**Authors:** Konstantin A. Bikov, Götz Schuck, Peter A. Georgiev

**Affiliations:** 1 Department of Condensed Matter Physics and Microelectronics, Sofia University “St. Kliment Ohridski”, 1164 Sofia, Bulgaria; bikov@phys.uni-sofia.bg; 2 Department of Structure and Dynamics of Energy Materials, Helmholtz-Zentrum Berlin für Materialien und Energie, Hahn-Meitner-Platz 1, 14109 Berlin, Germany; goetz.schuck@helmholtz-berlin.de

**Keywords:** nitric oxide, adsorption, thermodynamics, microporosity, gas storage, EXAFS

## Abstract

We studied the adsorption thermodynamics and mechanism behind the binding of nitric oxide (NO) in the interior surfaces and structural fragments of the high metal center density microporous Metal-Organic Framework (MOF) CPO-27-Cu, by gas sorption, at a series of temperatures. For the purpose of comparison, we also measured the corresponding CO_2_ adsorption isotherms, and as a result, the isosteric heats of adsorption for the two studied adsorptives were derived, being in the range of 12–15 kJ/mol for NO at loadings up to 0.5 NO molecules per formula unit (f.u.) of the bare compound (C_4_O_3_HCu), and 23–25 kJ/mol CO_2_ in the range 0–1 CO_2_ per f.u. Microscopically, the mode of NO binding near the square pyramid Cu(II) centers was directly accessed with the use of in situ NO gas adsorption X-ray Absorption Spectroscopy (XAS). Additionally, during the vacuum/temperature activation of the material and consequent NO adsorption, the electronic state of the Cu-species was monitored by observing the corresponding X-ray Near Edge Spectra (XANES). Contrary to the previously anticipated chemisorption mechanism for NO binding at Cu(II) species, we found that at slightly elevated temperatures, under ambient, but also cryogenic conditions, only relatively weak physisorption takes place, with no evidence for a particular adsorption preference to the coordinatively unsaturated Cu-centers of the material.

## 1. Introduction

Nitric oxide (NO), apart from its negative reputation as a toxic gas, and more generally as an environmental pollutant, is a well-known vasodilator and hemostasis promoter [1], also possessing strong antimicrobial [2,3] and wound-healing [4] properties. Quite astonishingly, some Gram-positive bacteria have developed bacterial nitric oxide synthases (bNOS) as a protective shield against antibiotics [5]. From a fundamental point of view, the NO molecule has long been considered as a probe molecule for various metal ion species on surfaces or extra-framework ions in zeolites, able to differentiate between their different oxidation states, e.g., Cu(I) and Cu(II) species, notwithstanding the recognized importance of nitric oxide as a main or intermediate species in environmentally important catalytic de-N_x_O_y_ processes [6,7].

**Figure 1:**
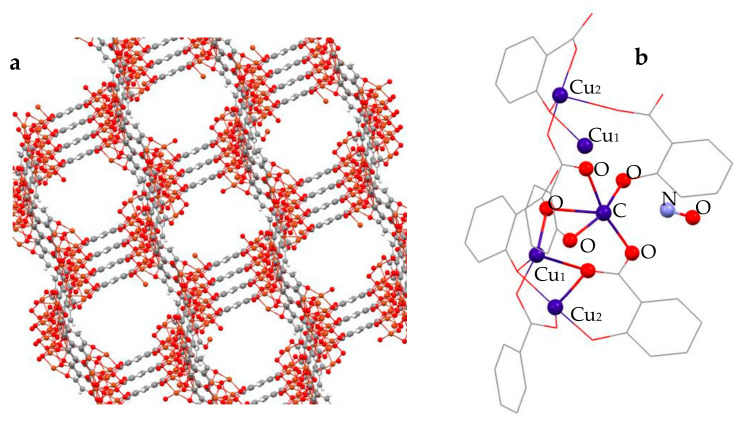
The micropore structure of CPO-27-Cu in (**a**,**b**) a cluster cut out from the corresponding hexagonal Bravais unit cell-based supercell, representing the chain sequence of Cu(II) coordinatively unsaturated centers as potential adsorption sites that were specifically probed by XAS. Structures visualized with Mercury 4.0 [31].

Various means are used to deliver and apply NO via organic and hybrid organic–inorganic chemical compounds which release NO in the environment of their application, e.g., nitroglycerin, nitrates, and nitroprusside [1]. Another type of material considered as a potential NO carrier is microporous compounds like zeolites, which are stable under a very broad range of environmental conditions, e.g., temperature and pH, and are biologically safe (being rather neutral) at the same time [8]. Metal-organic frameworks (MOFs) [9,10], on the other hand, are relatively new hybrid organic–inorganic materials, with a tunable reticular structure allowing for very diverse topologies, including microporosity of different size and accessible to guest species in varied chemical environments. Owing to these properties, a broad range of functionalities have been identified, with MOFs having been considered as materials for gas/vapor or more generally volatile molecular species capture, storage, and utilization [11,12,13], gas sieving and purification [14,15], catalysis [16], optical materials [17,18], and the delivery and controlled release of bioactive species for medicinal applications [19,20,21,22]. Similarly, NO capture by MOFs and targeted release have been the recent focus of scientific research [23,24,25]. The physicochemical nature of NO binding, at specific structural centers in MOFs, e.g., framework metal ions like Ni(II) [26] and Cu(II) [27], that largely determines the material’s applicability, has been studied with the use of in situ FTIR techniques and computationally by DFT techniques [28]. These studies confirm relatively strong chemical binding to Ni(II) [28], and moderate to weak binding to Cu(II) [27,28]. DFT simulations predict rather weak binding of NO at the square pyramidal coordinatively unsaturated Cu(II) centers in the CPO-27-Cu(II) MOF (also known as MOF-74(Cu)), but very strong (up to 90 kJ/mol) NO binding at the Ni(II) centers in the same MOF structure [28]. The case of NO binding and recovery [27] from the HKUST-1 Cu(II)-based framework [29] is intriguing, where out of 3 mmol adsorbed NO, corresponding to 1 NO/Cu center, only 2.2 mmol are desorbed back at ambient conditions and about 0.8 mmol appear strongly bound and trapped in the material [27]. Our earlier combined FTIR and computational DFT-based studies on the binding of H_2_, CO, and NO to the Cu-containing CPO-27-Cu MOF [30] (Figure 1) suggested that three and/or four oxygen atom-coordinated defect Cu(I) species may exist and be responsible for the strong binding of NO, in contrast to much weaker NO-binding at the regular pentacoordinated (square pyramidal) Cu(II) sites [30]. A similar scenario might take place in the HKUST-1 material too [27]. In an attempt to clarify these phenomena of clearly significant practical importance, we performed in situ XANES/EXAFS studies on the binding of NO at the Cu-centers of the CPO-27-Cu materials, which provide direct microscopic access to the local geometry. These studies, supplemented by direct NO gas volumetric sorption measurements at ambient conditions, are the focus of the present work, which is aimed at answering the question of what is the nature of the Cu(II)-NO binding, monitoring the electronic state of the acting Cu-species in situ.

## 2. Results and Discussion

**Figure 2:**
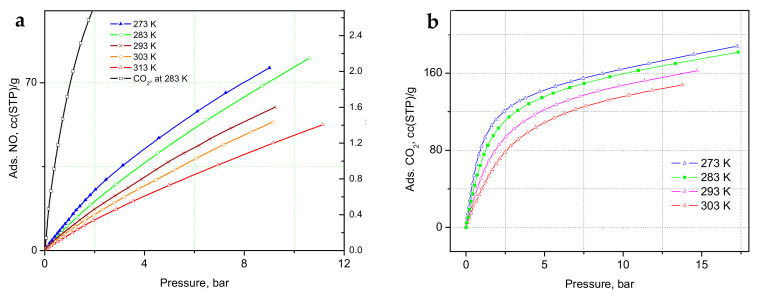
(**a**) Nitric oxide adsorption isotherms at a series of temperatures near ambient, along with a CO_2_ adsorption isotherm at 283 K for comparison; (**b**) Carbon dioxide adsorption isotherms in the same temperature range as indicated in the figure legends.

**Figure 3:**
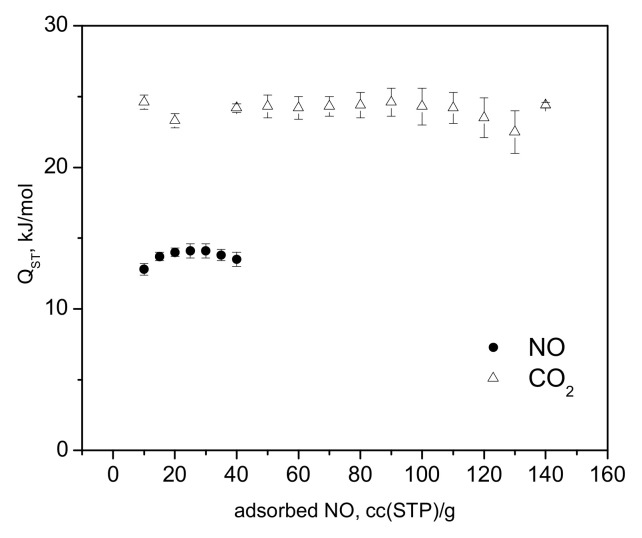
The isosteric heats of adsorption for carbon dioxide and nitric oxide in the CPO-27-Cu materials as derived from the adsorption isotherm data presented in Figure 2.

The adsorption isotherms of NO and CO_2_ measured at a range of temperatures are shown in Figure 2a,b. In the a-block of the figure, we compare the adsorption of CO_2_ at 283 K to the NO-sorption isotherms, where a much steeper gain in CO_2_ adsorption is clear, indicating a substantially higher heat of adsorption, with stronger binding of CO_2_ at these relatively low loadings, in total corresponding to only partial occupancy of the unsaturated Cu(II) sites [32,33]. The adsorbed amount of NO in the CPO-27-Cu material at 10 °C, (Figure 2a) is about 76 cc (STP)/g, just exceeding the loading corresponding to 0.5 adsorbate molecules per Cu(II) center. The same amount for CO_2_ at 283 K is adsorbed at only about 1.16 bar, in good agreement with earlier studies [32,34]. However, both the in situ CO_2_ neutron scattering study [32] and the synchrotron X-ray study [33] confirm that at room temperature, in contrast to the other members of the CPO-27(MOF-74) family, with other TM(II) metal centers, there is no adsorption site preference to the Cu(II) centers. The adsorbate is rather randomly distributed over at least three different adsorption sites in the pore interiors of the framework, including the metal sites, even at relatively low fractional loadings, i.e., well below the capacity of the metal sites alone. Hence, a similar adsorption scenario of NO at ambient temperatures should be expected considering the isotherm data in Figure 2a, and especially that derived from the above data isosteric heats of adsorption, Qst, shown in Figure 3, being almost a factor of two smaller than that for CO_2_, ranging between 12 ± 0.4 and 15 ± 0.5 kJ/mol for NO vs. 22 ± 0.5 to 25 ± 1.4 kJ/mol for CO_2_.

The latter is in good agreement with the earlier estimate [32], being close also to the enthalpy of sublimation of CO_2_, ca. 26 kJ/mol. To the best of our knowledge, the heats of adsorption of NO in CPO-27-Cu (MOF-74(Cu)) have not previously been reported.

**Figure 4:**
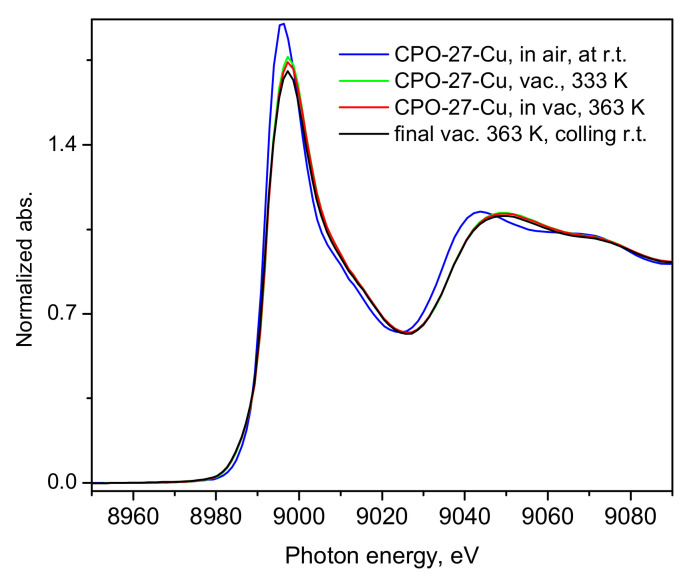
XANES spectra of the CPO-27-Cu material under different conditions as described in the figure legend. The last spectrum was collected after the adsorbed NO was evacuated at 363 K, during dynamic vacuum and the sample temperature was near the ambient.

**Figure 5:**
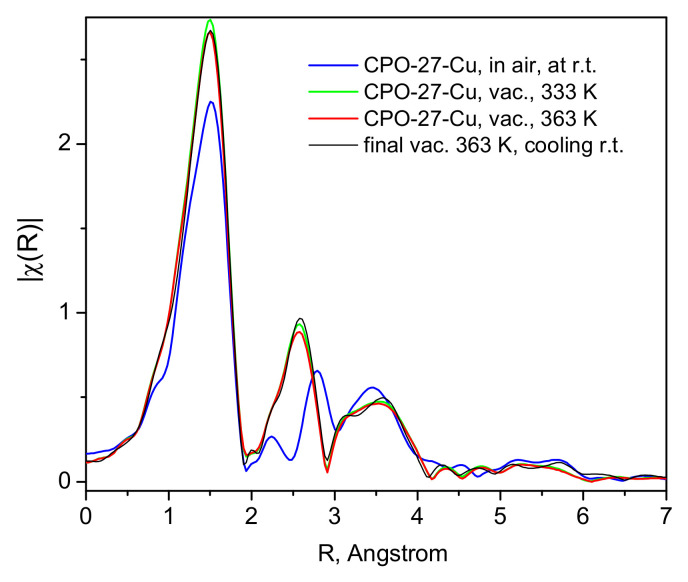
The corresponding EXAFS spectra collected during the activation of the material in vacuum and up to 363 K (after 3 h), as shown in the figure legend.

While it has already been unequivocally clarified for the case of CO_2_ [32,33], such low NO adsorption heats (Figure 3) clearly indicate that physisorption is the main mechanism responsible and likely the only uptake mechanism. Even if some small amount of defect Cu(I) sites do exist in our material, these would most likely remain blocked at the relatively low activation temperatures applied in the present study and hence would not contribute to the adsorption process. Turning to the microscopic point of view, the activation of the CPO-27-Cu material under a dynamic vacuum and mild heating up to 363 K was followed by recording the XANES/EXAFS spectra around the copper K-edge. These data, namely, the spectra recorded from the as-prepared in air tablet, after degassing at 333 K and 363 K (spectra measured at these temperatures), are shown in Figure 4 and Figure 5.

The shape and position of the Cu K-edge line in Figure 4 evidences the divalent state of the framework Cu-centers, which undergo very little, rather insignificant changes upon applied vacuum heat treatment, clearly leaving the oxidation state of the probed ion unchanged, with a small coordinative change due to the desorption of water and other air species picked up by the sample during its handling in air.

**Figure 6:**
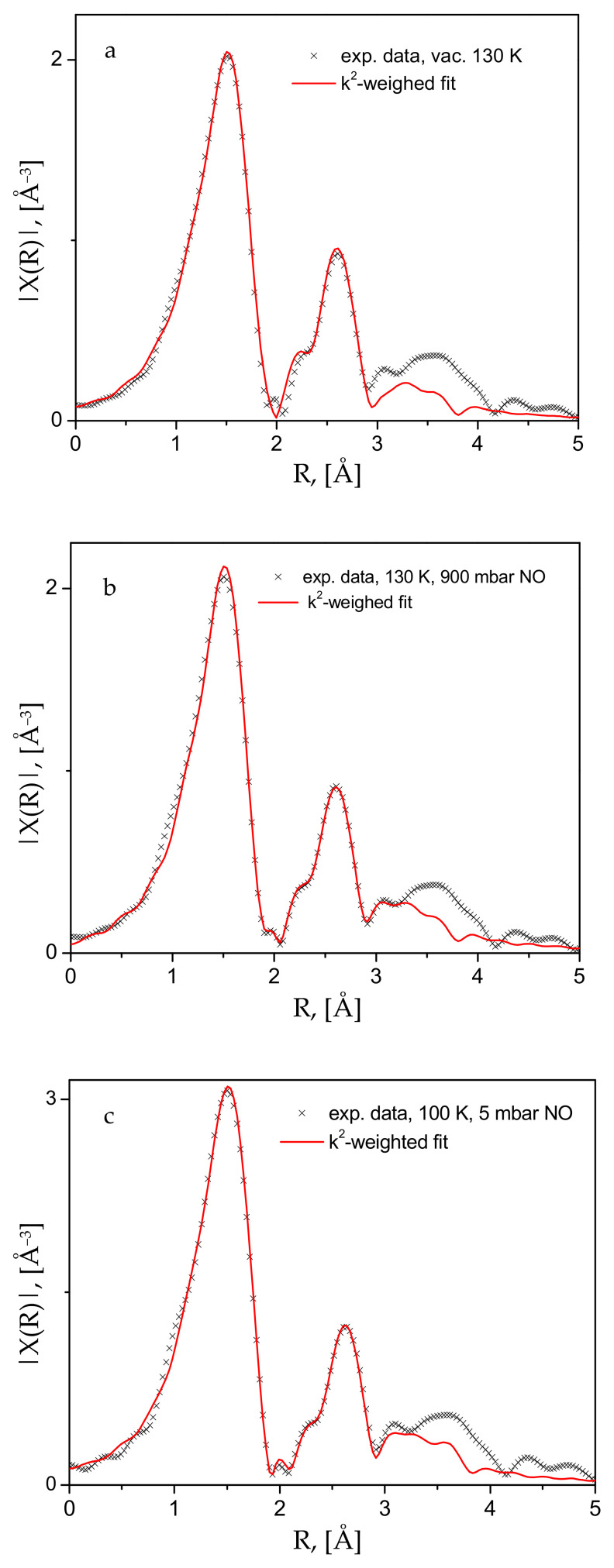
Experimental data and the corresponding fits to the k^2^-weighted Fourier-transformed EXAFS function for (**a**) the activated compound, cooled down to 130 K; (**b**) the same under an equilibrium pressure of 900 mbar; and (**c**) further cooled to 100 K, with the gas pressure reduced to 5 mbar.

These structural changes are observed in the EXAFS data (Figure 5), where the Fourier-transformed EXAFS function’s dependence on the distance to the scattering center is depicted for the bare material in air and at room temperature, and the same after evacuation at 333 and 393 K. The most noticeable change is the disappearance of the small peak at about 2.25 Å, replaced by a more intense feature at about 2.5 Å. The analyses of these data, performed with the aid of the Demeter package (version 0.9.26) [35], show unequivocally that the lowest distance most intense peak in Figure 5 originates from the Cu(II)-O scattering paths in the first coordination sphere. The peak at 2.25 Å must correspond to the signal from Cu(II) with various adsorbate guests, mostly H_2_O and CO_2_, but no satisfactory fits could be obtained for this spectrum. However, the fits of the other two spectra, i.e., in a dynamic vacuum at 333 and 363 K (Figure S3) and the activated sample spectrum at 130 K (Figure 6a and Figure S4 in the Supplementary Materials file), clearly show that the next peak is due to Cu-Cu scattering. To ensure better quality of the data and complete population of the Cu-centers by NO adsorbate, we also measured the spectra at low temperatures, e.g., 100–130 K. As seen in Figure 6, satisfactory fits to the data are achieved up to about 3.2 Å, a range which fully covers the first coordination sphere of the Cu-centers. The corresponding essential fit parameters and the resulting geometries of the studied structures are summarized in Table 1. The corresponding fitted χ(k) data, along with more model fit details are provided in the Supplementary Materials Figures S4–S7. Tables S1–S12 contain the complementary full fit and model structural details. Our data, both the XANES-evidenced electronic structure and the local Cu(II)-structure from the EXAFS fits, clearly show that the interactions between the divalent copper centers in the CPO-27-Cu MOF material and the guest NO molecules are of a physisorption nature—weak such that the adsorbate remains positioned at relatively large distances of ca. 2.5 Å (the Cu-N distance).

**Figure 7:**
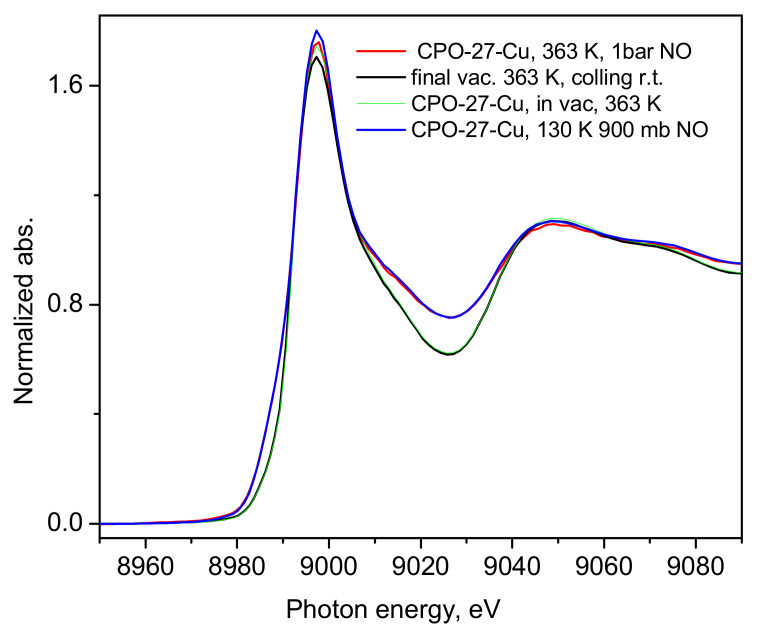
The XANES spectrum of the high temperature 1 bar NO loading—red line, compared to the spectrum of the first 900 mbar NO loading—blue line, after its corresponding first activation in a dynamic vacuum at 363 K—green line, and the final measurement of the spectrum under a dynamic vacuum at 363 K—black line.

Although the experimental set up used did not allow for determination of the adsorbed amount of NO, considering the magnitude of the adsorption heat calculated from our ambient conditions sorption isotherm data, we may assume that under the XAS experimental conditions, near and well below the normal boiling point of nitric oxide, 121 K, all the Cu-centers should be saturated, considering too that the adsorbate Q_st_ is a few kJ/mol larger than the corresponding heat of vaporization of NO (13.8 kJ/mol, NIST Chemistry WebBook, SRD 69), i.e., the MOF-NO interactions are somewhat stronger than the NO-NO interactions in the bulk liquid state. As a final test, we warmed the sample up to 363 K and again increased the gas pressure up to 1 bar to check if certain chemical interactions would follow due to thermal activation. This did not lead to any noticeable changes in the corresponding spectra, which are shown in Figure 7, compared to the earlier data discussed above.

Additionally, the state of the material, after the sorption measurements was checked by means of SEM. A corresponding image is shown in Figure S8, confirming the good crystalline shape of the material, along with the EDS analysis in Figure S9, indicating the expected Cu-content. Thus, the present XANES/EXAFS and NO gas isothermal adsorption studies may be regarded as a comprehensive test of the nature of the interactions between square pyramidal Cu(II) species and nitric oxide. Indeed, our XANES data (Figure 4 and Figure S1) and other recent studies on the bimetallic Co-Cu- [36] and Ni-Cu- [37] analogs of the same compound, show the presence only of the white line of Cu(II), with no sign of significant pre-edge features, which in the same compound do appear above 423 K [34]. According to our experience, however, the structure crystallinity deteriorates at these temperatures, followed by framework collapse, which may well lead to partial reduction of Cu(II) during the change in the nearest coordination environment. In contrast to earlier general assumptions [6,7] and references therein, our results show that with the pentacoordinate Cu(II) centers in the studied framework, nitric oxide interacts only weakly, remaining in the physisorption regime, as predicted by our earlier hybrid DFT-based computational studies [28,30] and as observed for carbon dioxide in earlier studies [32,33]. The structural stability in our experiments was checked after completion of the isotherm measurements by means of SEM and TGA (Figures S8–S11), showing the micron-size hexagonal cross-section crystals as previously observed [38]. Intriguingly, the TGA profile of the material suggests that it re-hydrated quite quickly upon exposure to air so as to consequently lose about 30% weight as expected at temperatures near 100 °C [38] (Figures S3 and S10). The fast adsorption kinetics observed in our isotherm studies, on the other hand, represent indirect evidence of the weak interactions between the Cu(II) centers and the guest molecules, which for this reason see a rather smooth pore interior facilitating the penetration and fast access to the material internal surfaces and surface sites. The present experimental results should not be generalized as valid for any Cu(II) species. We cannot exclude some stronger interactions involving different types of coordination of Cu(II) species, with either a lower coordination number and/or better exposure of the metal ion in defect states, zeolites or on oxide surfaces, for instance. While most practical applications of MOFs for NO storage and continuous spontaneous or stimulated delivery rely on chemical binding of NO, usually to inorganic SBUs of material, as recently reviewed in [39], the CPO-27-Cu material offers only physically adsorbed NO species, although of very similar quantities, e.g., 3 mmol NIO per gram adsorbent [39], which are instantly lost upon reduction in the NO pressure in the gas phase.

## 3. Experimental Procedures

CPO-27-Cu was synthesized according to the published procedure [38]. Nitric oxide adsorption isotherms were measured with a custom-built Sievert-type volumetric gas rig equipped with 2 bar and 20 bar pressure sensors with 0.04% accuracy (full scale, GE, UNIK 5000). NO gas of 99.8% purity was supplied by Linde gas Bulgaria EOOD, Sofia, Bulgaria. Carbon dioxide of purity 99.999% from Messer Bulgaria EOOD, Sofia, Bulgaria, was used as a comparator to the NO sorption studies. A sample of 0.43 g dry powder was evacuated prior to the adsorption measurements in a dynamic vacuum, first at room temperature for about 1 h and then stepwise increasing the temperature over a period of about 1 h up to 363 K, evacuating at this point for an additional 2 h. Isotherms were determined at a series of temperatures between 273 and 313 ± 0.1 K using a thermostat liquid bath (Argo Lab CB5-30, Giorgio Bormac s.r.l, Modena, Italy), allowing for a 15 min pressure equilibration time at each adsorption step. In practice, a few minutes were sufficient for most of the pressure change to equilibrate at each step, despite the large micron-sized sample crystallites [38] (see also the presented SEM images in the Supplementary Materials file accompanying this article). After the measurement of each adsorption isotherm, the sample was reactivated under similar conditions. The total desorbed amount of NO gas was checked down to 10 mbar desorption pressures, differing by under 2 cc (STP) compared to the measured total adsorbed amount. Sample reactor dead volumes were measured with He gas of 99.999% purity at several temperatures in the same range. Using the modified Clapeyron–Clausius Equation (1), the corresponding isosteric heats of adsorption, *Q_st_*, were derived from the isotherm data.

(1) $$\ln p=-\frac{1}{T}\frac{Q_{st}}{R}+const$$

XANES/EXAFS X-ray absorption studies were performed at the Berlin photon source BESSY II, on the KMC-2 beam line [40], at Helmholtz Zentrum, Berlin, Germany. In situ measurements were performed with the aid of specially designed at the BESSY II institute dome capped gas cell with Kapton windows, on a 12 mg powder sample compressed into a tablet form, at a 1:1 mass ratio CPO-27-Cu_x_H_2_O:BN, held between two Kapton foils. XANES/EXAFS spectra were measured from the as-prepared tablet in air and at room temperatures. Then the dome space was evacuated using a turbomolecular pump, while stepwise increasing the temperature up to 363 K, limited by the dome windows design, where the temperature was held constant for 3 h, while simultaneously recording the spectra. This temperature is, however, well beyond the 335 K at which complete dehydration of the CPO-27-Cu framework occurs [41]. The temperature was then decreased down to 130 K. The NO dosing was performed at a pressure of 160 mbar and the corresponding XAS spectra were measured after allowing 30 min equilibration time. The pressure was then increased up to 900 mbar and the corresponding spectrum was again measured after allowing for the equilibration period. The temperature was then reduced down to 100 K and the sample was equilibrated again for about 30 min at this temperature. The gas pressure in the cell was then reduced to 5 mbar, and the corresponding XAS spectra were measured. The system was then warmed up to 363 K and under 1 bar of NO pressure another XANES spectrum was measured to check for possible temperature activated processes. Following this, the sample was evacuated at this temperature for about 3 h. XAS spectra were consequently re-measured near room temperature to check the final sample state. More details, including the data analyses are provided in the Supplementary Materials information file.

## 4. Summary and Conclusions

Using gas in situ X-ray absorption techniques, combined with gas adsorption isotherm measurements, we studied the specific Cu(II) MOF host–NO guest interactions underlying the adsorption mechanism in CPO-27-Cu material. More specifically, at the microscopic level, we probed the electronic state and local coordination environment of the framework Cu(II) coordinatively unsaturated centers, at ambient conditions and after dynamic vacuum treatments up to 363 K, following complete dehydration of the materials. Furthermore, we monitored the oxidation state of the Cu(II)-centers upon activation and dosing of NO gas at a few different pressures up to 1 bar, at low temperatures, around the NBP of NO, e.g., 100 K, 130 K, as well as at 363 K, and observed changes in their first coordination sphere, but with no significant effect on the oxidation state. Our studies clearly show that the relatively low isosteric heat of adsorption determined from the variable temperature sorption isotherm data, ca. 16 kJ/mol, at low coverage, is consistent with a pure physisorption mechanism as far as the Cu(II) centers are concerned. At the low temperatures of the XAS studies, adsorbate molecules are found adsorbed at a ca. 2.5 Å distance from the probed Cu-ions, leading to no observable change in the adsorption site oxidation state, which we monitored through the observation of the corresponding XANES spectra. Notably, addressing previous spectroscopic and computational studies, hybrid DFT functional-based computational studies on the interaction of this paramagnetic molecule with open shell species like Cu(II) and Ni(II), for instance, correctly predict the nature and the magnitude of the interactions involved. Our findings emphasize the importance of the coordination type, along with the formal oxidation state of accessible Cu-species in microporous materials like MOFs and zeolites and should help the design of novel Cu-based catalytic but also NO storage and delivery systems for medicinal applications in which Cu(II) could be replaced by other metal ions. Alternatively, the binding site could be made more exposed to guest molecules either by further lowering its coordination number or pushing it out of the coordinating ligands plane.

## Figures and Tables

**Table 1:** Local structural data, extracted from the EXAFS fits and presented in Figure 6 (see Supplementary Materials for more detailed information).

Sample	Path	N (Fixed)	r [Å]	σ^2^ [Å^2^]	R-Factor
130 K, evacuated	Cu-O	2	1.922 (11)	0.005 (1)	0.023
	Cu-O	2	1.974 (11)	0.005 (1)	
	Cu-Cu *	2	2.977 (14)	0.007 (2)	
130 K, 160 mb NO	Cu-O	3	1.938 (8)	0.005 (1)	0.019
	Cu-O	1	1.960 (8)	0.005 (1)	
	Cu-N	1	2.489 (19)	0.005 (1)	
	Cu-Cu *	2	2.977 (11)	0.006 (1)	
130 K, 900 mb NO	Cu-O	3	1.939 (66)	0.005 (1)	0.016
	Cu-O	1	1.962 (66)	0.005 (1)	
	Cu-N	1	2.571 (46)	0.005 (1)	
	Cu-Cu *	2	2.9802 (98)	0.006 (1)	
100 K, 5 mb NO	Cu-O	3	1.944 (5)	0.0059 (8)	0.014
	Cu-O	1	1.967 (5)	0.0059 (8)	
	Cu-N	1	2.5395 (49)	0.0067 (9)	
	Cu-Cu *	2	2.995 (7)	0.00698 (87)	

* Cu-Cu is the scattering path between the central Cu-ion and the nearest Cu_1_-center along the helical chain, as depicted in Figure 1b, while the Cu-Cu_2_ distance is outside the fitting range.

## Data Availability

The raw data supporting the conclusions of this article will be made available by the authors on request.

## Supplementary Materials

The following supporting information can be downloaded at: https://www.mdpi.com/article/10.3390/molecules30143007/s1. Figures S1–S10 as well as Tables S1–S12 provide additional information and data treatment details regarding the analyses of the XANES and EXAFS spectra χ(k), scanning electron microscopy images of the CPO-27-Cu material taken after the sorption measurements and brief exposure to air, as well as thermal gravimetric analysis of the same material.
