# Supplementary material for: Reversible Binding of Nitric Oxide in a Cu(II)-Containing Microporous Metal-Organic Framework
Source: Molecules. 2025 Jul 17;30(14):3007. doi: 10.3390/molecules30143007 (PMC12298806; doi:10.3390/molecules30143007)
Supplement: Supplementary file 1 [file molecules-30-03007-s001.zip › molecules-3731054-supplementary.pdf]

Supplementary Information for:

Reversible binding of Nitric Oxide in a Cu(II)-containing microporous Metal-Organic Framework

Konstantin A. Bikov [1], Götz Schuck [2] and Peter A. Georgiev [1,*]

[1] Department of Condensed Matter Physics and Microelectronics, Sofia University "St. Kliment Ohridski", 1164 Sofia, Bulgaria; bikov@phys.uni-sofia.bg

[2] Department of Structure and Dynamics of Energy Materials, Helmholtz-Zentrum Berlin für Materialien und Energie, Hahn-Meitner-Platz 1, 14109 Berlin, Germany; goetz.schuck@helmholtz-berlin.de

* Correspondence: pageorgiev@phys.uni-sofia.bg

S1 Experimental XANES Spectra near the K-edge for Cu-CPO-27

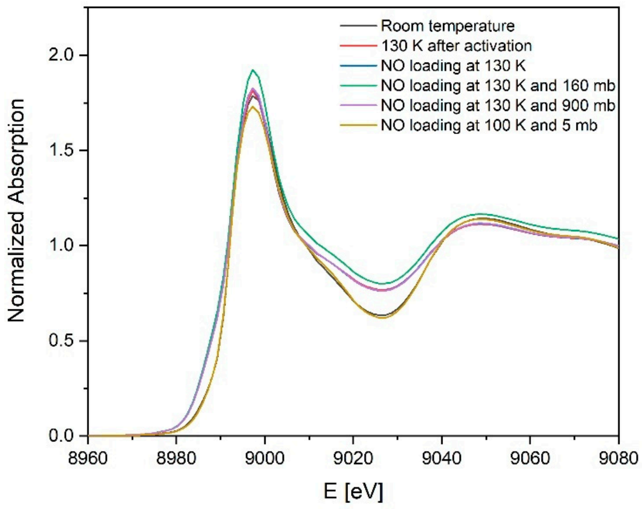

Figure S1 XANES spectra of Cu-CPO-27 under different measurement conditions

The XANES spectrum measured at room temperature clearly shows that the copper center in the MOF structure is in the second oxidation state, because a typical local maximum is observed at 8997 eV, followed by a steep decrease in absorption curve in the energy range of 9010 – 9030 eV. The addition of nitric oxide at different pressures and temperatures clearly does not change the structure and typical characteristics of the XANES spectrum.

S2 Experimental Fourier Transformed Extended X-ray Absorption Spectra (FT EXAFS Spectra) for Cu-CPO-27

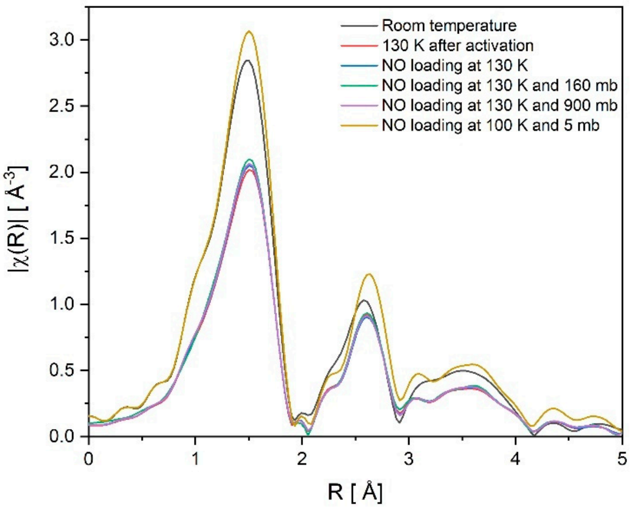

Figure S2 Experimental k^2-weighted Fourier transformed (FT) EXAFS spectra of Cu-CPO-27 under different measurement conditions

S3 EXAFS Analysis of measured absorption spectra at different temperature and pressure conditions

The initial structural model used in the EXAFS analysis was dft-optimized. Both single and multiple scattering paths were used in the fitting process, with the single photoelectron scattering paths having the most significant influence. The single scattering paths to the oxygen and carbon atoms were parameterized with independent interatomic distances and Debye-Waller factors. The carbon and oxygen atoms were assigned the same Debye-Waller factor in the fitting process, since the two types of atoms are not reliably distinguishable for EXAFS spectroscopy. The parameterization of the Debye-Waller factor for a given neighboring scattering atom is carried out by the following formula: $\sigma_i^2 = \sigma_0^2 (R_{eff,\,i} / R_0)^{1/2}$, where R_0 is the shortest distance to a given atom for a group of scattering paths, $R_{eff,i}$ is the distance to atom i, σ_0^2 is the independent parameter of the fit for the Debye-Waller factor for an atom located at a distance R_0 from the absorption center. In all fits, all coordination numbers N are fixed. Inflated values of S_0^2 are observed, which vary from 1.5 to 1.6. The reasons for this are probably due to the deteriorated quality of the EXAFS function $\chi(k)$ for values of the wavenumber k above 11 $Å^{-1}$. On the other hand, the fits of the experimental spectra at higher temperatures (333 K and 363 K) and at 100 K are distinguished by a value for S_0^2, which is too high and unphysical. The possible reason for observing such high values of S_0^2 may be the use of an insufficiently appropriate structural model.

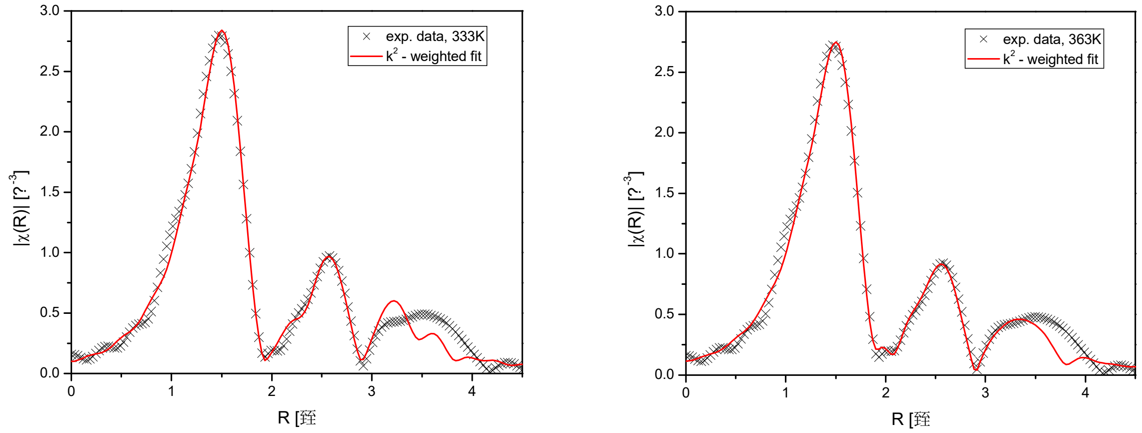

Figure S3 EXAFS data and model fits during the activation process in dynamic vacuum at 333 K, left, and 363 K – right block. The latter data were measured after about 3 hours at 363 K and dynamic vacuum.

S3.1 Absorption spectrum of Cu-CPO-27 at 333 K

Independent number of parameters N_{ind} = 21.5, number of parameters used N_{used} = 10.

Table S1 The parameters used in the fit to the data at 333 K, Fig. S3, left block.

S_0^2	ΔE [eV]	σ^2_{Cu-O} [Å^2]	σ^2_{Cu-Cu} [Å^2]	ΔR_{Cu-O} [Å]	ΔR_{Cu-O22} [Å]	ΔR_{Cu-Cu} [Å]	ΔR_{Cu-O12} [Å]
2.4(2)	-2.0(9)	0.0056(11)	0.011(2)	-0.040(7)	-0.18(3)	-0.097(13)	0.04(2)

ΔR_{Cu-O21}[Å]	$\Delta R_{Cu-O21-O13}$[Å]
0.18(9)	-0.06(4)

Table S2 Structural information extracted from the fit to the 333 K data. R-factor = 0.017.

N	Scattering Path	R_{eff} [Å] (from starting structure)	Interatomic distance R [Å]
2	Cu-O21	1.961	1.921(7)
2	Cu-O31	2.013	1.973(7)
1	Cu-O22	2.537	2.36(3)
1	Cu-C11	2.852	2.67(3)
3	Cu-C31	2.938	2.841(13)
1	Cu-O23	3.014	2.917(13)
2	*Cu-O11-C11*	3.052	*3.09(2)*
2	Cu-Cu	3.076	2.979(13)
2	*Cu-O21-C12*	3.087	*3.12(2)*
2	*Cu-O31-C32*	3.148	*3.18(2)*
1	Cu-C21	3.340	3.38(2)
1	Cu-C13	3.397	3.43(2)
1	Cu-C41	3.593	3.63(2)
1	Cu-O12	3.643	3.68(2)
2	*Cu-O21-O11*	3.901	*3.84(4)*
2	Cu-O21	3.922	4.10(9)
2	*Cu-O31-O32*	4.003	*3.94(4)*
2	Cu-O31	4.027	3.95(9)
2	Cu-O24	4.074	4.25(9)
2	*Cu-O21-O13*	4.128	*4.07(4)*
2	*Cu-C12-O13*	4.149	*4.09(4)*

S3.2 Absorption spectrum of Cu-CPO-27 at 363 K

Independent number of parameters N_{ind} = 21.5, number of parameters used N_{used} = 9.

Table S3 Fit parameters for the data at 363 K, Fig. S3, right block.

S_0^2	ΔE [eV]	σ^2_{Cu-O} [Å^2]	σ^2_{Cu-Cu} [Å^2]	ΔR_{Cu-O} [Å]	ΔR_{Cu-O22} [Å]	ΔR_{Cu-Cu} [Å]	ΔR_{Cu-C31}[Å]
2.5(2)	-0.64±0.79	0.0060(9)	0.014(4)	-0.036(6)	-0.20(3)	-0.137(15)	0.087(39)

ΔR_{Cu-C13}[Å]
0.11(5)

Table S4 Structural information extracted from the fit to the 363 K data. R-factor = 0.014.

N	Scattering Path	R_{eff} [Å] (from starting structure)	Interatomic distance R [Å]
2	Cu-O21	1.961	1.925(6)
2	Cu-O31	2.013	1.978(6)
1	Cu-O22	2.537	2.74(3)
1	Cu-C11	2.852	3.05(3)
3	Cu-C31	2.938	3.026(39)
1	Cu-O23	3.014	2.877(15)
2	*Cu-O11-C11*	3.052	*3.106 (fixed)*
2	Cu-Cu	3.076	2.939(15)
2	*Cu-O21-C12*	3.087	*3.142 (fixed)*
2	*Cu-O31-C32*	3.148	*3.202 (fixed)*
2	*Cu-O32-C31*	3.152	*3.206 (fixed)*
1	Cu-C21	3.340	3.428(39)
1	Cu-C13	3.397	3.50(11)
1	Cu-C41	3.593	3.70(11)
1	Cu-O12	3.643	3.85(2)
2	*Cu-O21-O11*	3.901	*3.898 (fixed)*
2	Cu-O21	3.922	4.12(2)
2	*Cu-O31-O32*	4.003	*4.001 (fixed)*
2	Cu-O31	4.027	4.13(11)
2	Cu-O24	4.074	4.18(2)
2	*Cu-O21-O13*	4.128	*4.125 (fixed)*
2	*Cu-C12-O13*	4.149	*4.146 (fixed)*

S3.3 Absorption spectrum of Cu-CPO-27 at 130 K after activation

Independent number of parameters N_{ind} = 20.9, number of parameters used N_{used} = 9.

Table S5 Used parameters in the fit

S_0^2	ΔE [eV]	σ^2_{Cu-O} [$Å^2$]	σ^2_{Cu-Cu} [$Å^2$]	ΔR_{Cu-O} [Å]	ΔR_{Cu-O22} [Å]	ΔR_{Cu-Cu} [Å]	ΔR_{Cu-C1} [Å]
1.6(2)	-1.44 ±1.53	0.005(1)	0.0071(16)	-0.039(11)	-0.16(3)	-0.098(14)	-0.09(3)

ΔR_{Cu-C2} [Å]
0.065(36)

Table S6 Structural information extracted from the fit. R-factor = 0.023.

N	Scattering Path	Reff [Å] (from starting structure)	Interatomic distance R [Å]
2	Cu-O21	1.961	1.922(11)
2	Cu-O31	2.013	1.974(11)
1	Cu-O22	2.537	2.38(3)
1	Cu-C11	2.852	2.76(3)
3	Cu-C31	2.938	2.85(3)
1	Cu-O23	3.014	2.92(3)
2	Cu-Cu	3.076	2.977(14)
2	*Cu-O21-C12*	3.087	*3.053 (fixed)*
1	Cu-C21	3.340	3.405(36)
1	Cu-C13	3.397	3.462(36)
1	Cu-C41	3.593	3.658(36)
1	Cu-O12	3.643	3.708(36)
1	Cu-C22	3.855	3.920(36)
2	Cu-O24	4.074	4.203(72)

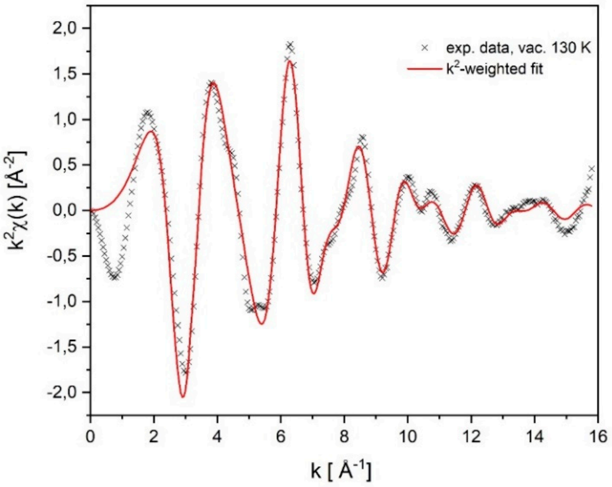

Figure S4 EXAFS fit in k-space at 130 K after activation

S3.4 Absorption spectrum of Cu-CPO-27 at 130 K and NO pressure of 160 mB

Independent number of parameters N_{ind} = 21.3, number of parameters used N_{used} = 10.

Table S7 Used parameters in the fit

S_0^2	ΔE [eV]	$\sigma^2_{Cu\text{-}O}$ [Å^2]	$\sigma^2_{Cu\text{-}Cu}$ [Å^2]	$\Delta R_{Cu\text{-}O}$ [Å]	$\Delta R_{Cu\text{-}O22}$ [Å]	$\Delta R_{Cu\text{-}Cu}$ [Å]	$\Delta R_{Cu\text{-}O11\text{-}C11}$ [Å]	$\Delta R_{Cu\text{-}N11\text{-}O41}$ [Å]	$\Delta R_{Cu\text{-}O21\text{-}O13}$ [Å]
1.58(16)	-2.39±1.31	0.0049(11)	0.005996(1161)	0.0024(78)	-0.041(19)	-0.0065(108)	0.157(67)	0.053(72)	-0.033(105)

Table S8 Structural information extracted from the fit. R-factor = 0.019.

N	Scattering Path	Reff [Å] (from starting structure)	Interatomic distance R [Å]
3	Cu-O21	1.935	1.938(8)
1	Cu-O32	1.958	1.960(8)
1	Cu-O22	2.411	2.370(19)
1	Cu-N11	2.530	2.489(19)
1	Cu-C11	2.774	2.733(19)
1	Cu-C31	2.835	2.794(19)
1	Cu-C32	2.876	2.835(19)
1	Cu-O23	2.881	2.840(19)
1	Cu-C12	2.919	2.878(19)
2	Cu-Cu	2.9835	2.977(11)
2	*Cu-O11-C11*	3.014	*3.171(67)*
2	*Cu-O32-C31*	3.056	*3.213(67)*
2	*Cu-O31-C32*	3.063	*3.220(67)*
2	*Cu-O21-C12*	3.070	*3.226(67)*
1	Cu-C21	3.284	3.440(67)
1	Cu-C13	3.332	3.488(67)
1	Cu-O41	3.409	3.566(67)
2	*Cu-N1-O41*	3.542	*3.595(72)*
1	Cu-O12	3.604	3.657(72)
4	*Cu-O21-O11*	3.857	*3.910(72)*
4	Cu-O21	3.882	3.935(72)
2	*Cu-O21-O13*	4.131	*4.099(105)*
1	Cu-O24	3.936	3.989(72)
1	Cu-O13	4.081	4.238(67)
1	Cu-C14	4.112	4.269(67)

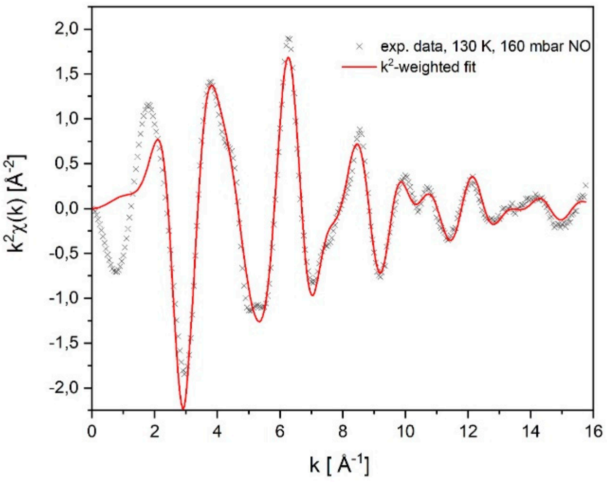

Figure S5 EXAFS fit in k-space at 130 K and under pressure of 160 mB

S3.5 Absorption spectrum of Cu-CPO-27 at 130 K and NO pressure 900 mB

Independent number of parameters N_{ind} = 21.7, number of parameters used N_{used} = 10.

Table S9 Used parameters in the fit of the data

S_0^2	ΔE [eV]	σ^2_{Cu-O} [Å^2]	σ^2_{Cu-Cu} [Å^2]	ΔR_{Cu-O} [Å]	ΔR_{Cu-O22} [Å]	ΔR_{Cu-Cu} [Å]	ΔR_{Cu-N11} [Å]
1.50(13)	-0.81±1.03	0.0047(10)	0.0062(12)	0.0040(66)	-0.039(13)	-0.0033(98)	0.041(46)
$\Delta R_{Cu-N11-O41}$ [Å]	$\Delta R_{Cu-O11-C11}$ [Å]						
0.042(68)	0.156(59)						

Table S10 Structural information extracted from the fit. R-factor = 0.016.

N	Scattering Path	Reff [Å] (from starting structure)	Interatomic distance R [Å]
3	Cu-O21	1.935	1.939(66)
1	Cu-O32	1.958	1.962(66)
1	Cu-O22	2.411	2.372(13)
1	Cu-N11	2.530	2.571(46)
1	Cu-C11	2.774	2.856(92)
1	Cu-C31	2.835	2.796(13)
1	Cu-C32	2.876	2.837(13)
1	Cu-O23	2.881	2.803(26)
1	Cu-C12	2.919	3.000(92)
2	Cu-Cu	2.9835	2.9802(98)
2	*Cu-O11-C11*	3.014	3.170(56)
2	*Cu-O32-C31*	3.056	3.212(56)
2	*Cu-O31-C32*	3.063	3.219(56)
2	*Cu-O21-C12*	3.070	3.226(59)
1	Cu-C21	3.284	3.440(59)
1	Cu-C13	3.332	3.489(59)
1	Cu-O41	3.409	3.565(59)
2	*Cu-N1-O41*	3.542	3.583(68)
1	Cu-O12	3.604	3.646(68)
4	*Cu-O21-O11*	3.857	3.899(68)
4	Cu-O21	3.882	3.923(68)
1	Cu-O24	3.936	3.978(68)
2	Cu-O21-O13	4.131	4.106 (fixed)
2	Cu-C12-O13	4.152	4.127 (fixed)
2	Cu-C11-C23	4.183	4.158 (fixed)

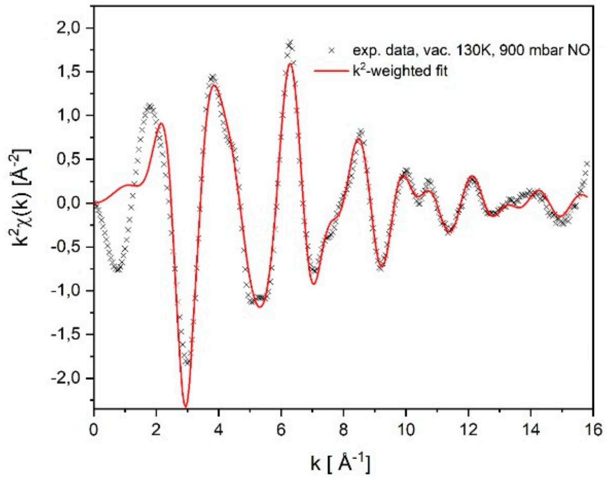

Figure S6 EXAFS fit in k-space at 130 K and under 900 mb NO pressure.

S3.6 Absorption spectrum of Cu-CPO-27 at 100 K and NO pressure of 5 mb

Independent number of parameters N_{ind} = 21.7, number of parameters used N_{used} = 9.

Table S11 Used parameters in the fit

S_0^2	ΔE [eV]	σ^2_{Cu-O} [Å^2]	σ^2_{Cu-Cu} [Å^2]	ΔR_{Cu-O} [Å]	ΔR_{Cu-O22} [Å]	ΔR_{Cu-Cu} [Å]	ΔR_{Cu-C21} [Å]
2.65(19)	-0.77±0.68	0.0059(8)	0.00698(87)	0.0092(49)	-0.097(15)	0.012(7)	0.093(57)

$\Delta R_{Cu-O11-C11}$ [Å]
0.001(5)

Table S12 Structural information extracted from the fit. R-factor = 0.014.

N	Scattering Path	Reff [Å] (from starting structure)	Interatomic distance R [Å]
3	Cu-O21	1.935	1.944(5)
1	Cu-O32	1.958	1.967(5)
1	Cu-O22	2.411	2.314(15)
1	Cu-N11	2.530	2.5395(49)
1	Cu-C11	2.774	2.783(5)
1	Cu-C31	2.835	2.844(5)
1	Cu-C32	2.876	2.885(5)
1	Cu-O23	2.881	2.783(15)
1	Cu-C12	2.919	2.928(5)
2	Cu-Cu	2.9835	2.995(7)
2	*Cu-O11-C11*	3.014	3.015(5)
2	*Cu-O32-C31*	3.056	3.057(5)
2	*Cu-O31-C32*	3.063	3.064(5)
2	*Cu-O21-C12*	3.070	3.070(5)
1	Cu-C21	3.284	3.377(57)
1	Cu-C13	3.332	3.425(57)
1	Cu-O41	3.409	3.502(57)
2	*Cu-N1-O41*	3.542	3.542(5)
1	Cu-O12	3.604	3.697(57)
4	*Cu-O21-O11*	3.857	3.858(5)
4	Cu-O21	3.882	3.975(57)
2	Cu-O21-O13	4.131	4.132(5)
2	Cu-C12-O13	4.152	4.152(5)
2	Cu-C11-C23	4.183	4.184(5)

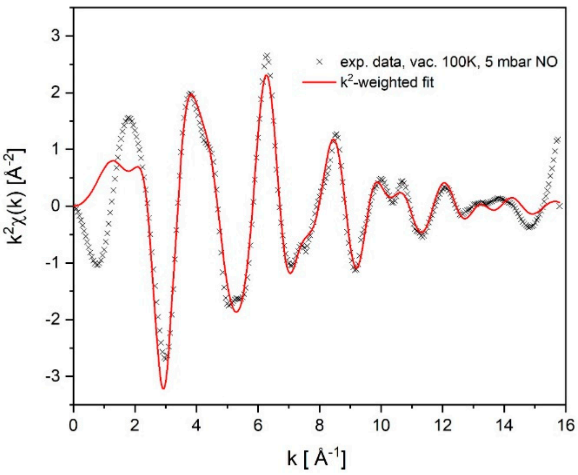

Figure S7 EXAFS fit in k-space at 100 K and under pressure 5 mB

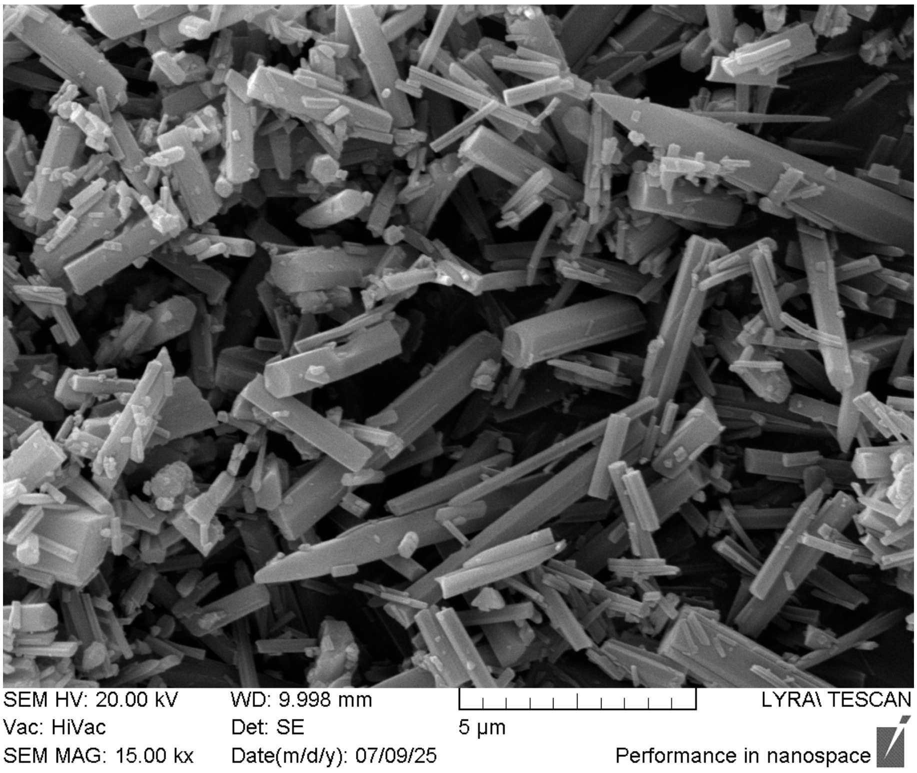

Figure S8. SEM image(TESCAN Lyra I XMU) of the crystalline CPO-27-Cu powder, taken after the NO and CO_2 sorption measurements, and after exposure to air for about a day prior to the microscopy.

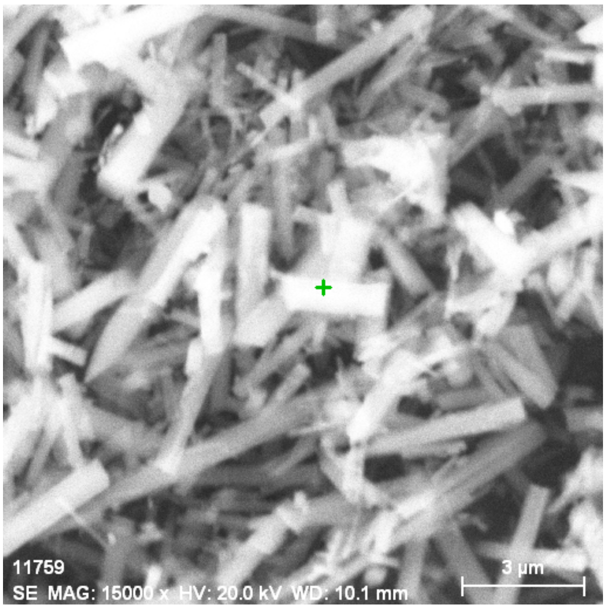

11759Date:7/9/2025 12:13:23 PMImage size:512 x 512Mag:15000xHV:20.0kV

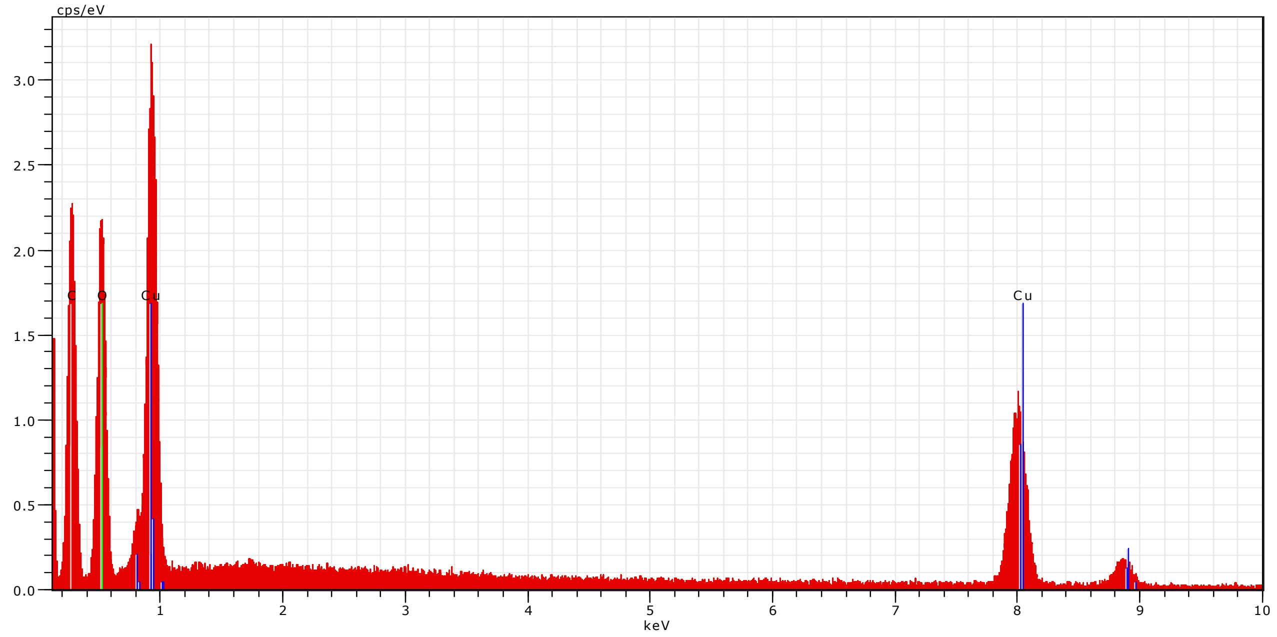

Acquisition 12725 Date:7/9/2025 12:05:04 PM HV:20.0kV Puls th.:1.57kcps

El	AN	Series	unn. C [wt.%]	norm. C [wt.%]	Atom. C [at.%]	Error [%]
C	6	K-series	20.57	20.03	33.40	2.7
O	8	K-series	45.37	44.19	55.32	5.9
Cu	29	K-series	36.74	35.78	11.28	1.1
		Total:	102.68	100.00	100.00	

Figure S9 Energy Dispersive x-ray Spectroscopic (EDS, Quantax 200, Bruker) analysis of the material, taken at the green spot shown in the image above, indicating the expected Cu-content in CPO-27-Cu.

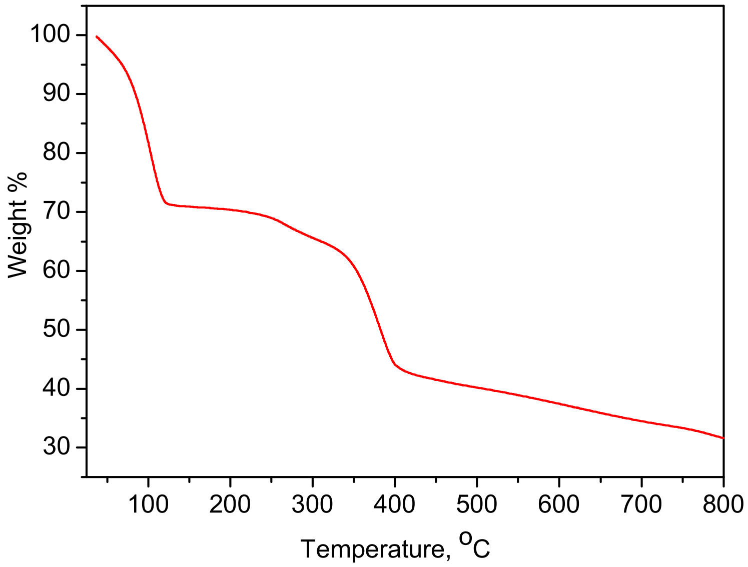

Figure S10. Thermal Gravimetric Analysis of the CPO-27-Cu material measured after the sorption isotherm measurements and brief exposure to air, in argon 5.0 purity, using a Perkin Elmer DSC-8500 instrument. The temperature scan speed was 5ºC/min from room temperature up to 400ºC/min, then 10ºC/min.
